# A ten-year study of midwife-led care at an Austrian tertiary care center: a retrospective analysis with special consideration of perineal trauma

**DOI:** 10.1186/s12884-017-1544-9

**Published:** 2017-10-16

**Authors:** Barbara Bodner-Adler, Oliver Kimberger, Julia Griebaum, Peter Husslein, Klaus Bodner

**Affiliations:** 1 Department of Obstetrics and Fetomaternal Medicine, Medical University of Vienna, Währinger Gürtel 18–20, 1090 Vienna, Austria; 2 Department of Anesthesiology, Medical University of Vienna, Wien, Austria

**Keywords:** Midwife-led care, Doctor-led care, Low-risk maternity, Perineal trauma

## Abstract

**Background:**

In contrast to other countries, Austria rarely offers alternative models to medical led-care. In an attempt to improve the facilities, a midwife-led care service was incorporated within the Department of Obstetrics and Fetomaternal Medicine. The aim of the present study was to analyze the maternal and neonatal outcomes of this approach.

**Methods:**

Over a 10-years period, a total of 2123 low-risk women receiving midwife-led care were studied. Among these women, 148 required obstetric referral. Age- and parity matched low-risk women with spontaneous vaginal birth overseen by an obstetrician-led team were used as controls to ensure comparability of data.

**Results:**

Midwife-led care management demonstrated a significant decrease in interventions, including oxytocin use (*p* < 0.001), medical pain relief (*p* < 0.001), and artificial rupture of membranes (ARM) (*p* < 0.01) as well as fewer episiotomies (*p* < 0.001), as compared with obstetrician-led care. Moreover, no negative effects on maternal or neonatal outcomes were observed. The mean length of the second stage of labor, rate of perineal laceration and APGAR scores did not differ significantly between the study groups (*p* > 0.05). Maternal age (*p* < 0.01), head diameter (*p* < 0.001), birth weight (p < 0.001) and the absence of midwife-led care (*p* < 0.05) were independent risk factors for perineal trauma. The overall referral rate was low (7%) and was most commonly caused by pathologic cardiotocography (CTG) and prolonged first- and second-stage of labor. Most referred mothers nevertheless had spontaneous deliveries (77%), and there were low rates of vaginal operative deliveries and cesarean sections (vacuum extraction, 16%; cesarean section, 7%).

**Conclusions:**

The present study confirmed that midwife-led care confers important benefits and causes no adverse outcomes for mother and child. The favorable obstetrical outcome clearly highlights the importance of the selection of obstetric care, on the basis of previous risk assessment. We therefore fully support the recommendation that midwife-led care be offered to all low-risk women and that mothers should be encouraged to use this option. However, to increase the numbers of midwife-led care deliveries in Austria in the future, it will be necessary to expand this care model and to establish new midwife-led care units within hospital facilities.

## Background

Maternity care in Austria is mainly hospital-based and consultant-led. As it is in other European countries, this public, in-hospital medical service is covered by a general, compulsory health insurance and is provided free of charge to all pregnant women [1, 2]. The obstetrician’s involvement and responsibility is predominant, even in normal childbirth, whereas the influence of midwives in the hospital setting – although they have the legal right to practice completely autonomously and unsupervised – tends to be limited.

The routine use of various interventions such as episiotomy, electronic fetal monitoring and pain control has strongly increased during recent years [3–5], although such interventions are not recommended in the the guidelines of the World Health Organization (WHO) on the care of women giving birth, nor have they been demonstrated to improve maternal or neonatal outcomes.

Despite recommendations supported by multiple societies like the United Kingdom (UK) National Institute for Health and Clinical Excellence (NICE), The Royal Australian and New Zealand College of Gynaecologists and Obstetricians (RANZCOG), The New Zealand College of Midwives (NZCOM) and the Royal College of Obstetricians and Gynaecologists (RCOG) a continuous electronic fetal monitoring of low-risk pregnancies is still performed in the majority of patients [2, 6–9]. While maternal and neonatal mortality rates have decreased in developed countries over the past decades for either medical or social reasons, the medical supervison of labor and delivery in low-risk women has long surpassed its efficiency [2, 10, 11]. Widespread fear of litigation has led to an increase in unnecessary interventions and operative deliveries, which both contribute to a rise in maternal and neonatal morbidity and affect the healthy relationship between mother and child [12–16].

This unfavorable trend represents new challenges for health professionals aiming to avoid unnecessary treatment and to promote normalcy during low-risk pregnancy and childbirth [17]. The selection of obstetric care based on risk assessment has been suggested by previous research, and many countries have made a strong effort to implement alternative models of care for low risk-women to improve outcomes for mother and child [3, 18–21].

Worldwide midwife-led settings have been shown to be associated with decreased medical interventions and increased maternal satisfaction [22]. In Austria, in contrast to other countries, such as the Netherlands or UK, alternative models to medical-led care are still rarely available. Likewise, studies on the topic of midwife-led care are lacking in Austria.

In an attempt to improve the Austrian healthcare facilities, a midwife-led service has been initiated within the Department of Obstetrics and Fetomaternal Medicine at the Medical University of Vienna and offers its care to all low-risk maternity women willing to use this option. Preliminary data with promising results regarding this approach have previously been published by the authors [23].

The aim of the present study was to extend the analysis to a period of more than 10 years and to record the maternal and neonatal outcomes of this midwife-led care service.

## Methods

At the Department of Obstetrics and Fetomaternal Medicine of the University Hospital of Vienna, approximately 2600 women on average give birth annually. The tertiary care level of this department is technically well equipped and staffed for handling severe medical situations during labor and delivery. In contrast to systems in other countries, the Austrian system allows low-risk women to register for birth at the University Hospital if capacity is sufficient. Their delivery then occurs under the guidance and responsibility of an obstetrician-led team.

In 1997, a midwife-led service was implemented that is offered to all women at low risk of complications. This service is, however, not separated from the conventional delivery ward and uses the same rooms for labor and birth. The midwife-led care team consists of 21 certified and experienced midwives who agreed to and felt confident in participating in this project.

In spite of the hospital setting, these midwives try to strive for normal birth processes with minimal intervention in labor. Partners and other support persons are encouraged to take an active role in physical and emotional support. Measures to help manage pain, such as movement, massage, shower and bathtube and pharmacological pain relief with opiates are available. Pregnant women are selected at birth registration according to their own preference for delivery if they meet the conditions for a midwife-led birth. Responsible midwives discuss participation with every interested woman and an informed written consent is obtained in each case.

Women must be generally healthy with uncomplicated pregnancies, and the babies must not have any congenital malformation or fetal/placental disease. Women are primarily excluded from the midwife-led service if any of the following characteristics are recorded: multiple gestation, pregnancy with non cephalic presentation, history of cesarean section, gestational age < 37^th^ weeks, known fetal macrosomia or retardation, other maternal risk factors such as diabetes mellitus or hypertension, history of intrauterine fetal death, premature rupture of the membranes and desire for epidural analgesia during delivery.

During the entire study period, over more than one decade, 2123 midwife-led low-risk women were studied, 148 of whom had to be referred to an obstetrician. To ensure the comparability of data, each mother was matched, on the basis of the delivery record book, with an age- and parity-identical low-risk woman who experienced spontaneous vaginal birth under care of an obstetrician-led team.

During the entire observation period, the patients’ background data were collected from the pregnancy and delivery records by using a computer-assisted database. Variables selected to analyze the outcome of the two services included the following maternal outcomes: maternal age, parity, gestational age, length of first and second stage of labor, amniotomy, use of oxytocin to augment labor, the use of medical pain relievers, maternal birth position, episiotomy, perineal trauma, severe postpartum hemorrhage (defined as maternal blood loss > 500 ml) and postpartum infection. A postpartal maternal infection was diagnosed mainly on the basis of clinical parameters such as maternal pyrexia, subinvolution of the uterus, illsmelling discharge and the need for intravenous antibiotical treatment. Additional indicators of maternal infection were leucocytosis and elevated CRP levels. Infant parameters used were APGAR scores at 1 and at 5 min (applying a cut-off point of 7 as the minimal acceptable score) and arterial cord pH.

Perineal traumas were categorized by using the traditional definitions of first-, second-, and third-degree perineal tear [24]. According to the department’s policy, episiotomies were performed either midline or mediolateral.

The study protocol was approved by the ethics committee of the Medical University of Vienna (EK no 1293/2016). All patients’ records were anonymized and de-identified prior to analysis.

### Statistical analysis

Chi-square tests were used to compare the frequency distributions of binary outcome variables between the group of women with midwife-led service and the obstetrician-led control group. Continuous variables were compared by using a T-test. *P*-values < 0.05 were considered statistically significant. Univariate and multiple logistic regression models were used to evaluate the influence of delivery management and other potential risk factors on perineal injury. The SPSS system (IBM, Armonk, NY, USA, Version 23) was used for the calculations.

## Results

### Obstetric referrals

During an observation period of more than 10 years, data from 4098 women were recorded. Among the mothers under midwife-led care, 148/2123 (7%) had an obstetric referral for various reasons (Table 1). A pathologic CTG was the most common cause for referral to an obstetrician (62/148; 42%). Prolonged first- and second-stage of labor was also a prevalent reason for terminating midwife-led care (37/148; 25%). Thirteen women requested epidural analgesia (13/148; 9%), and 16 mothers (16/148; 11%) required manual placental removal in the third stage of labor. In thirteen out of 148 women (11%), no obvious referral reason was recorded. Intrapartum hypotonia and patients’ decision caused cessation of midwife-led care in one case. The majority of referred mothers could be delivered spontaneously (114/148; 77%). The rates of vacuum extraction and cesarean section were 24/148 (16%) and 10/148 (7%), respectively.

**Table 1:** Total number and reasons for obstetric referral

	*n* = 148	100%
Reasons	n	
Pathologic CTG	62	42%
Prolonged first/s stage	37	25%
Unclear	18	12%
Hypotonia	1	1%
Womans‘decision	1	1%
Epidural analgesia	13	9%
Manual removal of placenta	16	11%
Mode of delivery among referrals	n	%
Spontaneous vaginal delivery	114	77%
Cesarean section	10	7%
Vacuum extraction	24	16%

### Maternal outcomes

Characteristics of the midwife-led and obstetrician-led groups are shown in Table 2. Differences regarding the obstetric management and various aspects of labor and delivery are shown in Table 3.

**Table 2:** Characteristics of midwife-led and doctor-led group (*n* = 4098)

	Midwife-led	doctor-led	*p*-value
	*n* = 2123	*n* = 1975	
Maternal age^a^	28 (24–32)	28 (24–32)	> 0.05
Gestational age (weeks)^a^	40 (39–41)	40 (39–41)	> 0.05
Parity			
Primiparous	578 (27%)	515 (26%)	> 0.05
Multiparous	1545 (73%)	1460 (74%)	> 0.05

^a^median (25% and 75% quartile)

**Table 3:** Differences regarding the obstetric management during labor and delivery between midwife-led and doctor-led service (*n* = 4098)

	Midwife-led	doctor-led	*p*-value
	*n* = = 2123 100%	*n* = 1975 100%	
length first stage, minutes^a^			*p* < 0.001
	318 (307–328)	292 (284–301)	
length second stage, minutes^a^			*p* > 0.05
	72 (39–92)	76 (42–98)	
Artificial rupture of membranes			*p* < 0.01
Yes	237 11%	269 14%	
No	1836 87%	1644 83%	
Missing	50 2%	63 3%	
Oxytocin augmentation			*p* < 0.001
Yes	180 8%	360 18%	
No	1943 92%	1615 82%	
Medical pain relief			*p* < 0.001
Yes	1093 51%	1203 61%	
No	1030 49%	772 39%	
Birthing position			*p* < 0.001
Supine	1579 74%	1574 80%	
lateral recumbent	228 11%	154 8%	
Upright	270 13%	181 9%	
Water	46 2%	66 3%	
Maternal blood loss			*p* > 0.05
< 500 ml	2080 98%	1949 99%	
> 500 ml	42 2%	26 1%	
Missing	1 0%	0 0%	
Maternal Infection			*p* > 0.05
Yes	23 1%	32 2%	
No	2099 99%	1943 98%	
Missing	1 0%	0 0%	

^a^median (25%–75% quartile)

Midwife-led care, as compared with labor management by an obstetrician, was associated with significant decreases in oxytocin use (180/2123; 8% versus 360/1975; 18%, *p* < 0.001) and medical pain relievers (1093/2123; 51% versus 1203/1975; 61%, *p* < 0.001). The use of artificial rupture of membranes (ARM) was significantly less common among midwives compared with obstetricians (237/2123; 11% versus 269/1975; 14%, *p* < 0.01). The length of the first stage of labor was significantly prolonged in midwife-led care (*p* < 0.001), whereas the second stage of labor did not significantly differ between the groups (*p* > 0.05).

The majority of women in both groups delivered in the supine position. However, the upright birth position was significantly more common among the midwife-supervised deliveries (270/2123; 13% versus 216/1975; 9%, *p* < 0.001).

The rates of increased maternal blood loss (> 500 ml) in the third stage of labor and maternal postpartum infections were comparable between the groups (*p* > 0.05). The frequency and localization of perineal trauma are listed in Table 4. A statistically significant lower number of episiotomies was perfomed by midwives compared with obstetricians (183/2123; 9% versus 267/1975; 14%, *p* < 0.001). The frequency and severity of perineal tears as well as the rate of vaginal tears and labial trauma did not differ significantly between the groups (*p* > 0.05).

**Table 4:** Frequency and localization of perineal trauma among midwife-led and doctor-led women (*n* = 4098)

	midwife-led	doctor-led	*p*-value
	*n* = 2123 100%	*n* = 1975 100%	
Perineal tear			*p* > 0.05
First degree	381 18%	327 17%	
Second degree	141 7%	109 6%	
Third degree	13 0.6%	9 0.4%	
Vaginal tear	303 14%	277 14%	*p* > 0.05
Labial trauma	187 9%	178 9%	*p* > 0.05
Episiotomy			*p* < 0.001
None	1940 91%	1708 86%	
Midline	15 1%	22 1%	
Mediolateral	168 8%	245 13%	

### Neonatal outcome

Neonatal characteristics were comparable in both cohorts and are given in Table 5. The number of neonates with a cord ph < 7.1 as well as with APGAR scores <7 at 1, 5 and 10 min did not differ significantly between the study groups (*p* > 0.05; Table 5).

**Table 5:** Neonatal outcome among midwife-led and doctor-led deliveries (*n* = 4098)

	Midwife-led	doctor-led	*p*-value
	*n* = 2123 100%	*n* = 1975	100%
Weight (g)^a^	3400 (3100–3700)	3420 (3120–3720)	*p* > 0.05
Size (cm)^a^	51 (50–53)	51 (50–53)	*p* > 0.05
Head diameter (cm)^a^	34 (34–35)	34 (34–35)	*p* > 0.05
Shoulder diameter (cm)^a^	37 (36–39)	37 (36–39)	*p* > 0.05
APGAR score			
At 1 min <7	69 3%	41 2%	*p* > 0.05
At 5 min <7	16 0.7%	6 0.3%	*p* > 0.05
At 10 min <7	10 0.4%	8 0.4%	*p* > 0.05
Cord pH < 7.1	77 4%	93 5%	*p* > 0.05

^a^median (25%- 75% quartile)

### Risk factors for perineal trauma

To evaluate the effects of various other factors that have been associated with perineal trauma, univariate and multivariate logistic regression models were used. Absence of midwife-led care (*p* < 0.05), higher maternal age (*p* < 0.01), primiparity (*p* < 0.05), large head diameter (*p* < 0.05) and high birth weight (*p* < 0.001) were risk factors for perineal trauma in the univariate analysis. When the multivariate analysis was performed, age (*p* < 0.01), head diameter (*p* < 0.001), birth weight (*p* < 0.001) and the absence of midwife-led care (*p* < 0.05) remained independent risk factors for perineal trauma.

## Discussion

To the best of our knowledge, the present study is the most extensive analysis of midwife-led care in German-speaking countries. Moreover, it is the first report in which midwife-led care service was directly incorporated into the facilities of a tertiary care center.

The main finding of our study was that midwife-led care, even in the highly sophisticated system of a tertiary care level hospital, produced significant positive effects on physiological outcomes for women. In particular, high rates of spontaneous vaginal deliveries with low numbers of obstetric referrals and reduced numbers of medical interventions were observed among the women receiving midwife-led care. The results included low rates of operative deliveries in the referral group, decreased oxytocin use to augment labor, decreased use of pain relievers and a lower frequency of artificial rupture of membranes (ARM) and episiotomies. Nevertheless, no negative effects on maternal and neonatal outcomes were detected.

Similarly to some other countries, Austria is experiencing growing trend of medical interventions [2]. The number of cesarean sections performed is considerably higher than that in some other European countries, such as Sweden, Norway and France, yet the health of the mother or the child has not improved [2, 25].

Nearly all women prefer to give birth in hospitals, and options for maternity service other than medical-led care are not as widely established in Austria as in other countries, such as the Netherlands and UK [25]. Thus, women with either high-risk or low-risk pregnancies have the option to register for birth at the tertiary care center of the Medical University of Vienna. An additional midwife-led care service was established within the Department of Obstetrics and Fetomaternal Medicine, to improve the facilities and to relieve the doctors’ workload within this highly specialized institution and allow doctors to focus on women with more complex needs.

Although this service is not infrastructurally separated and shares the same rooms and ward with labor and delivery, midwives treat these low-risk women autonomously and without supervision by the usual obstetrician-led team on call. We believe that, one major advantage of this incorporated setting is safety; in the case of any complications, an immediate referral with local treatment by the obstetrician on call is possible. We believe that safety during labor and delivery is one of the greatest concerns for every woman during childbirth. Therefore, the most powerful argument for the high acceptance of this new approach was the secure, close collaboration between the midwife-led care service and the conventional hospital-based system, thus ensuring mothers’ and infants’ safety during the entire birth process.

The results of our analysis were consistent with findings from most previous research [26, 27]. In accordance with the literature, midwife-led care was also found to have positive effects on some physiological maternal outcomes [26, 28, 29]. One remarkable finding was the high rate of spontaneous vaginal deliveries and low number of referrals and operative interventions among women with midwife-led care. Among the 2123 low risk-women in the midwife-assisted cohort, only 148 needed to be referred to the obstetrician on call, and a total of 10 cesarean sections and 24 vaccuum extractions were performed. This favorable outcome with an operative intervention rate far below the Austrian average of 30% clearly highlights the importance of the selection of obstetric care on the basis of previous risk assessment.

To date several advantages of restricted use of episiotomy have been proposed in literature and routine use of episiotomy is no longer supported by scientific evidence [30]. In particular since the 1996 World Health Organization recommended an episiotomy rate of approximately 10%, rates of episiotomy have been generally in decline [31]. For example, in the United States the episiotomy rate dropped from 17.3 to 11.6% from 2006 to 2012 [32]. The present study found episiotomy rates of 9% and 14%, respectively, with significantly lower numbers during midwife-led care. This finding is in accordance with those of previous investigators [28, 33–36]. Furthermore, the low episiotomy rate was also accompanied by low overall rates of perineal trauma indicating the excellent perineal outcome of the present trial.

Moreover, and in accordance with findings from previous investigations, the midwife-led treatment was also associated with other benefits, such as decreased number of interventions, including ARM, use of oxytocin to augment labor and use of pharmological pain relievers [28, 33–36].

Nevertheless, no adverse effects on maternal or neonatal health were found in the present observations. In line with results from the literature, the mean length of the second stage of labor, the rates of perineal lacerations and postpartum hemorrhage as well as APGAR scores showed no evidence of a difference between the two obstetric care providers [29, 33, 35].

We are aware that, owing to the retrospective study design, definitive conclusions cannot be drawn, and further prospective randomized studies are necessary to evaluate this midwife-led care model. However, the obstetrical outcome of the present trial was excellent and deserves the further attention of policy makers and health care providers, particularly because it represents an important counterpart to the recent unfavorable developments in hospitals with increasing intervention rates, even for low-risk births.

Due to the observed physiological benefits with reduced numbers of interventions comprising low rates of episiotomies, vacuum deliveries, caesarean sections, medical pain relief, etc. midwife-led service seems to be the more-cost effective model of delivery service – even in the absence of a robust cost factor analysis. Furthermore, as also previous studies found that indicators of lower costs of midwife-led care are emerging [37, 38]. This is in fact a relevant issue especially in times of global economic crisis with a shortage of health budgets in most countries.

Currently, the availability of different options for maternity service is limited, and alternatives to the medical-led care model are rare in Austria. Our study shows, that the midwife-led care service of the Medical University of Vienna is an interesting, safe and effective birthing alternative to the highly medicalized obstetrician-led care.

## Conclusions

The present study confirmed that midwife-led care confers important benefits and causes no adverse outcomes for mother or child. We therefore support the recommendation that midwife-led care should be offered to all low-risk women and that mothers should be encouraged to use this option. However, to increase the numbers of midwife-led care deliveries in Austria in the future, it will be necessary to expand this care model and to establish new midwifery-led care units within existing hospital facilities.
